# Use of Endoscopic Ultrasound in a Diagnostic Dilemma: Metastatic Breast Cancer to the Stomach

**DOI:** 10.1155/2018/2820352

**Published:** 2018-04-15

**Authors:** Laura L. Ulmer, Ian Cormier, Lokesh K. Jha, Shailender Singh, Kurt W. Fisher, Alexander T. Hewlett

**Affiliations:** ^1^Department of Gastroenterology and Hepatology, University of Nebraska Medical Center, 982000 Nebraska Medical Center, Omaha, NE 68198-2000, USA; ^2^Department of Internal Medicine, University of Nebraska Medical Center, 42nd and Emile Street, Omaha, NE 68198-6805, USA; ^3^Internal Medicine Division of Gastroenterology-Hepatology, 982000 Nebraska Medical Center, Omaha, NE 68198-2000, USA; ^4^Department of Pathology and Microbiology, University of Nebraska Medical Center, 986805 Nebraska Medical Center, Omaha, NE 68198-6805, USA; ^5^Internal Medicine Division of Gastroenterology-Hepatology, University of Nebraska Medical Center, 982000 Nebraska Medical Center, Omaha, NE 68198-2000, USA

## Abstract

A 55-year-old woman presented with persistent nausea, vomiting, and weight loss previously attributed to Ménétrier's disease. On further workup, she was found to have metastatic lobular breast carcinoma causing gastric outlet obstruction, diagnosed by endoscopic ultrasound with fine needle aspiration after previous gastric mucosal biopsies were unremarkable. In most reported cases of gastric metastasis from breast cancer, a diagnosis was established by mucosal biopsy. This case is an important reminder that mucosal biopsies can be negative in about 20% of patients with gastric metastasis, which may lead to a delay in diagnosis and treatment. Gastroenterologists should be diligent in taking deeper biopsies if there is a suspicion for gastric metastasis.

## 1. Introduction

Though metastatic breast cancer to the stomach is rare, invasive lobular subtypes are known to metastasize to the gastrointestinal tract and can occur years after the initial cancer treatment [1–4]. Clinical manifestations can be vague with varying endoscopic and radiologic appearances, with diffuse infiltrating disease most commonly seen [5–7]. Endoscopic mucosal biopsies often confirm the diagnosis, though some patients require deep biopsies or surgical biopsies due to tumor infiltration of layers deep to the mucosa [8, 9].

## 2. Case Report

A 55-year-old woman with a history of invasive lobular breast cancer in 2009 treated with bilateral mastectomy, adjuvant chemotherapy/radiation, and tamoxifen therapy presented with ten weeks of nausea, vomiting, early satiety, and weight loss. She was diagnosed with Ménétrier's disease at another institution after esophagogastroduodenoscopy (EGD) revealed large gastric folds. Gastric biopsies demonstrated mild chronic inflammation, foveolar hyperplasia, and some glandular atrophy, though parietal and chief cell hypoplasia was not present. Her symptoms persisted despite initial supportive therapy and she presented to our institution for further evaluation.

**Figure 1:**
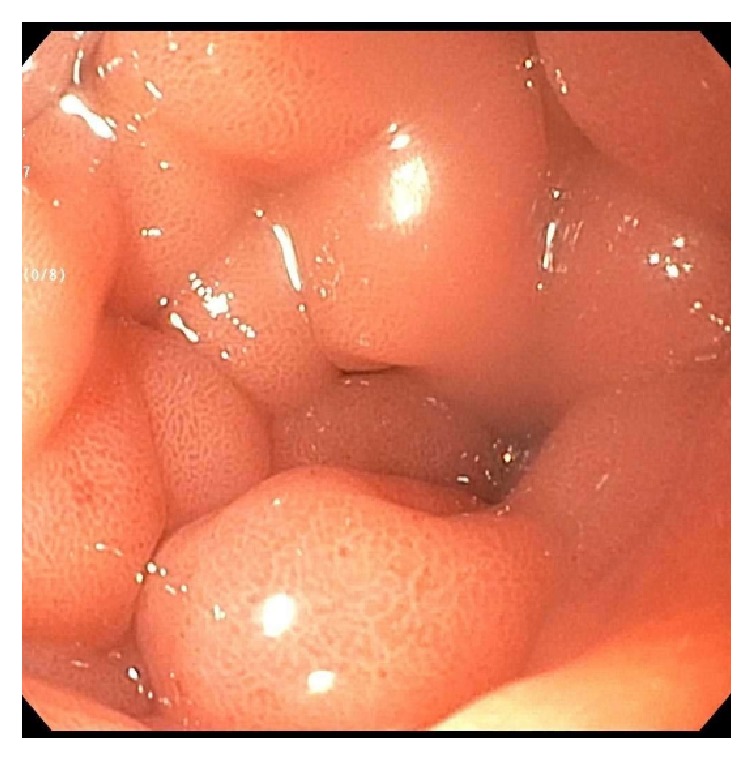
Enlarged prepyloric folds with luminal narrowing.

**Figure 2:**
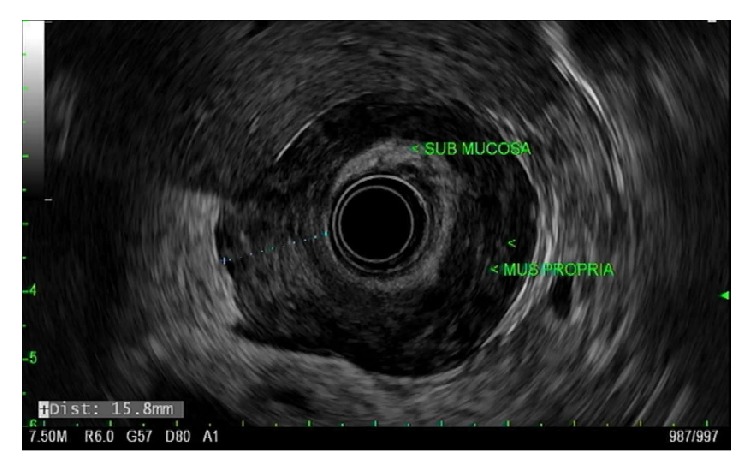
Thickened gastric muscularis propria layer on endoscopic ultrasound.

**Figure 3:**
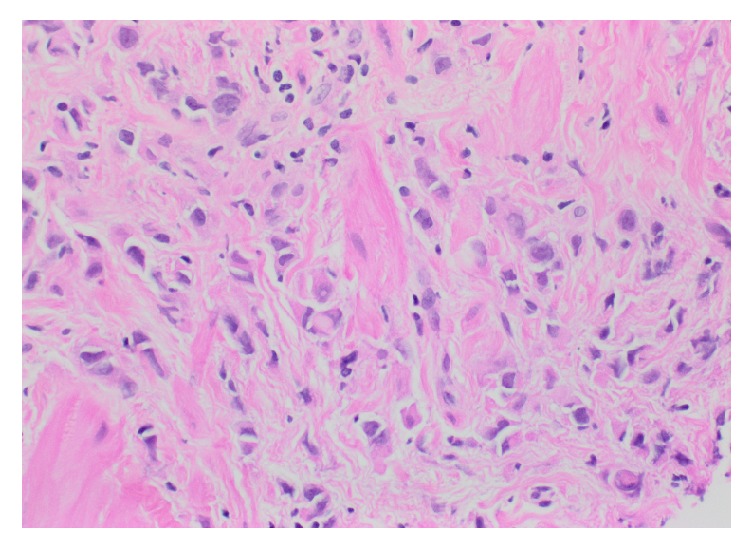
Hematoxylin and eosin (H&E) stain of FNA from gastric antrum showing tumor cells admixed with large pink bundles of muscle.

**Figure 4:**
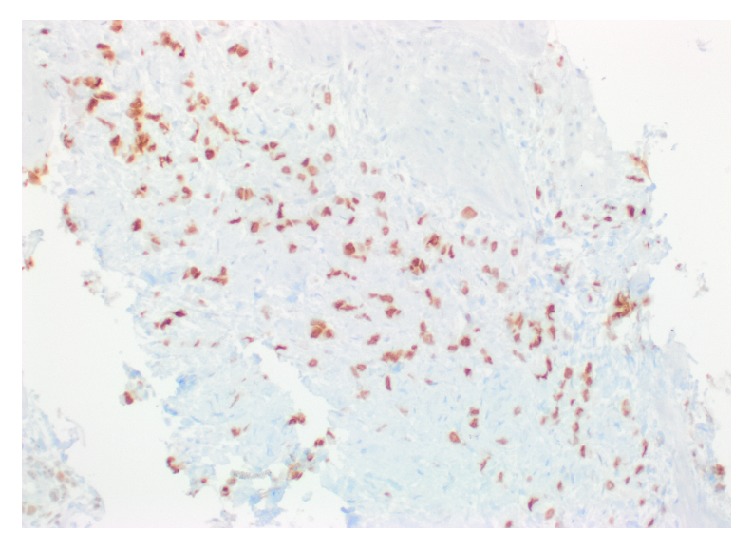
Immunohistochemical stain for GATA3, a lineage marker for breast origin.

**Figure 5:**
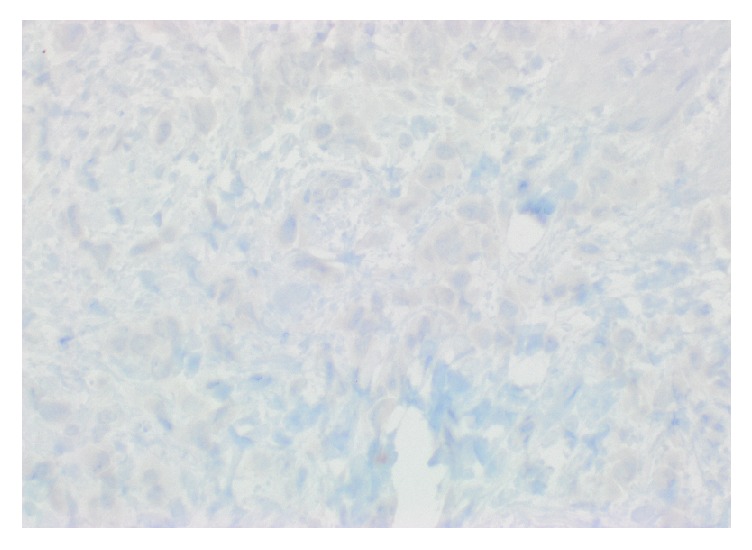
Immunohistochemical stain for CDX-2, a marker of gastrointestinal differentiation.

A repeat EGD revealed diffusely hypertrophied gastric folds, most prominent in the gastric antrum, with luminal narrowing of the pylorus (Figure 1). Biopsies from the gastric antrum and body showed mild chronic inactive gastritis. Given the lack of antral sparing of enlarged gastric folds as well as no reproducible evidence of foveolar hyperplasia, the diagnosis of Ménétrier's disease was thought to be unlikely. Further investigation with endoscopic ultrasound (EUS) showed marked concentric thickening of the muscularis propria in the gastric antrum (Figure 2) and fine needle biopsy (FNB) was performed with a 22-gauge Acquire™ EUS FNB needle. The specimen was preserved in formalin and received by the pathology department for immunostaining. Histology revealed infiltration of the gastric muscular wall by discohesive pleomorphic cells (Figure 3), positive for GATA3 (Figure 4) and negative for CDX2 (Figure 5) on immunohistochemistry, compatible with metastatic lobular breast carcinoma. A positron emission tomography scan demonstrated mildly metabolic diffuse wall thickening involving the gastric antrum, pylorus, and proximal duodenum. Magnetic resonance imaging of the brain and cerebrospinal fluid analysis were consistent with leptomeningeal carcinomatosis. The patient had palliative radiation therapy to her whole brain and gastric lesion. A palliative pyloric stent was placed with improvement of obstructive symptoms.

## 3. Discussion

Breast cancer is the most commonly diagnosed cancer and leading cause of cancer-related death among females worldwide. The incidence of breast cancer is higher in western, developed nations and lower in Africa and Asia [1]. Invasive lobular carcinoma (ILC) is the second most common subtype of breast cancer and represents approximately 10% of invasive breast carcinomas [2, 4]. Whereas the most common sites of metastases for invasive ductal carcinoma (IDC) are lung, liver, bone, and brain, ILC is known for its atypical metastatic pattern, which includes sites such as the gastrointestinal tract and peritoneum [10, 11]. It is important for the clinician to have a high index of suspicion for metastasis in patients with a history of breast cancer who present with vague gastrointestinal symptoms. Endoscopic ultrasound can be a useful diagnostic tool when mucosal biopsies are negative and can later be helpful in assessing the response of metastatic foci to chemotherapy when no other site is involved [5].

## References

[1] Almubarak M. M., Laé M., Cacheux W. (2011). Gastric metastasis of breast cancer: A single centre retrospective study. *Digestive and Liver Disease*.

[2] Borst M. J., Ingold J. A. (1993). Metastatic patterns of invasive lobular versus invasive ductal carcinoma of the breast. *Surgery*.

[3] Namikawa T., Hanazaki K. (2014). Clinicopathological features and treatment outcomes of metastatic tumors in the stomach. *Surgery Today*.

[4] Taal B. G., den Hartog Jager F. C., Steinmetz R., Peterse H. (1992). The spectrum of gastrointestinal metastases of breast carcinoma: II. The colon and rectum. *Gastrointestinal Endoscopy*.

[5] Lorimier G., Binelli C., Burtin P. (1998). Metastatic gastric cancer arising from breast carcinoma: endoscopic ultrasonographic aspects. *Endoscopy*.

[6] Rusticeanu M., Schuster M., Moga S. L. (2011). Metastatic lobular breast cancer presenting as gastric linitis plastica. *American Journal of Medicine*.

[7] Whitty L. A., Crawford D. L., Woodland J. H., Patel J. C., Nattier B., Thomas C. R. (2005). Metastatic breast cancer presenting as linitis plastica of the stomach. *Gastric Cancer*.

[8] Nazareno J., Taves D., Preiksaitis H. G. (2006). Metastatic breast cancer to the gastrointestinal tract: a case series and review of the literature. *World Journal of Gastroenterology*.

[9] Pectasides D., Psyrri A., Pliarchopoulou K. (2009). Gastric metastases originating from breast cancer: report of 8 cases and review of the literature. *Anticancer Reseach*.

[10] Arpino G., Bardou V. J., Clark G. M., Elledge R. M. (2004). Infiltrating lobular carcinoma of the breast: tumor characteristics and clinical outcome. *Breast Cancer Research*.

[11] Weigelt B., Peterse J. L., van't Veer L. J. (2005). Breast cancer metastasis: markers and models. *Nature Reviews Cancer*.

